# Influence of Cannabinoid Receptor Deficiency on Parameters Involved in Blood Glucose Regulation in Mice

**DOI:** 10.3390/ijms21093168

**Published:** 2020-04-30

**Authors:** Juliane Zibolka, Anja Wolf, Lisa Rieger, Candy Rothgänger, Anne Jörns, Beat Lutz, Andreas Zimmer, Faramarz Dehghani, Ivonne Bazwinsky-Wutschke

**Affiliations:** 1 Institute of Anatomy and Cell Biology, Medical Faculty of Martin Luther, University Halle-Wittenberg, 06108 Halle (Saale), Germany; juliane.zibolka@medizin.uni-halle.de (J.Z.); anja.wolf@medizin.uni-halle.de (A.W.); lisa.rieger2@student.uni-halle.de (L.R.); candy.rothgaenger@medizin.uni-halle.de (C.R.); faramarz.dehghani@medizin.uni-halle.de (F.D.); 2 Institute of Clinical Biochemistry, Hannover Medical School, 30625 Hannover, Germany; Joerns.Anne@mh-hannover.de; 3 Institute of Physiological Chemistry, University Medical Center of the Johannes Gutenberg University of Mainz, 55128 Mainz, Germany; beat.lutz@uni-mainz.de; 4 Institute of Molecular Psychiatry, Medical Faculty, University of Bonn, 53127 Bonn, Germany; a.zimmer@uni-bonn.de

**Keywords:** cannabinoid receptor knockout mice, endocannabinoid system, pancreatic islet, insulin, glucagon, somatostatin, glucose transporter, glucokinase

## Abstract

Cannabinoids are known to influence hormone secretion of pancreatic islets via G protein-coupled cannabinoid receptor type 1 and 2 (CB_1_ and CB_2_). The present study was designed to further investigate the impact of cannabinoid receptors on the parameters involved in insulin secretion and blood glucose recognition. To this end, CB_1_ and CB_2_ receptor knockout mice (10–12 week old, both sexes) were characterised at basal state and compared to wild-type mice. The elimination of cannabinoid receptor signalling resulted in alterations of blood glucose concentrations, body weights and insulin levels. Changes were dependent on the deleted receptor type and on the sex. Analyses at mRNA and protein levels provided evidence for the impact of cannabinoid receptor deficiency on the glucose sensing apparatus in the pancreas. Both receptor knockout mouse lines showed decreased mRNA and protein amounts of glucose transporters Glut1 and Glut2, combined with alterations in immunostaining. In addition, pancreatic glucokinase expression was elevated and immunohistochemical labelling was modified in the pancreatic islets. Taken together, CB_1_ and CB_2_ signalling pathways seem to influence glucose sensing in β-cells by affecting glucose transporters and glucokinase. These alterations were more pronounced in CB_2_ knockout mice, resulting in higher blood glucose and lower plasma insulin levels.

## 1. Introduction

Hormone secretion of pancreatic islet cells is influenced via G protein-coupled receptors (GPCRs) which are characterised by their seven transmembrane helical domains and their coupling to diverse intracellular signalling pathways [1]. GPCR function is receiving significant interest, since over 30 GPCRs have currently been implicated in the development and progression of beta-cell dysfunction, insulin resistance, obesity and type 2 diabetes mellitus, some of which have successfully been targeted therapeutically [2].

Cannabinoid receptors are GPCRs that have gained increased attention over recent years. Among them, cannabinoid receptors CB_1_ and CB_2_ were first described [3,4]. Cannabinoid receptors are part of the endocannabinoid system (ECS), and emerging evidence suggests an important role of these receptors in the pathogenesis of type 2 diabetes mellitus and its chronic complications [5]. In this context, CB_1_ and CB_2_ have been clearly identified in rodent and human pancreatic islets [6,7,8], as well as in diverse rodent beta-cell lines [9]. Until now, there has neither been a consensus about the distribution of these receptors in islet cell types within one species, nor between different species [10]. Measurements of downstream signalling upon cannabinoid receptor activation in the pancreatic islets with endogenous or pharmacological cannabinoids have led to conflicting results [7,10,11]. A reliable tool for the investigation of cannabinoid receptor functions in pancreatic islets became available after the development of single cannabinoid receptor knockout mouse lines: CB_1_-deficient mice [12,13] and CB_2_-knockout mice [14]. Previous observations on CB_1_^−/−^ mice revealed an absence of insulin resistance normally occurring in high fat diet-fed mice [15]. Mice deficient in CB_1_ displayed no changes in glucose tolerance and insulin sensitivity in association with diet-induced obesity [16]. Furthermore, in CB_2_^−/−^ mice, high fat diet-induced insulin resistance was reduced [17] and a lack of CB_2_-mediated responses also protected mice from both age-related and diet-induced insulin resistance [18]. Another study on diet-induced obesity in CB_2_-knockout and CB_1/2_ double-knockout mice reported that mice lacking both of the cannabinoid receptors were lean and resistant to diet-induced obesity. This phenotype was distinct from CB_2_-deficient mice which displayed signs of impaired glucose clearance [19]. Notably, in global models lacking cannabinoid receptors, other organs than pancreatic islets such as liver, skeletal muscle or adipose tissue might be directly or indirectly involved in the regulation of blood glucose levels or insulin resistance. In this context, many peripheral tissues are being influenced by the ECS controlling whole-body metabolism as shown in tissue-specific, genetically modified mice [20]. Still, only one study directly analysed the impact of CB_1_ receptor knockout on pancreatic islet function including insulin, glucokinase and glucose transporter 2 [21]. However, components of the glucose sensing machinery such as glucokinase and glucose transporters are essential for islet cell function [22,23]. Notably, glucose transporter 2 (Glut2) is required for glucose stimulated insulin secretion in rodent islets, while GLUT1 plays a major role in human beta-cells [23].

Thus, the present study was performed to investigate the contribution of each cannabinoid receptor deletion in the regulation of hormonal secretion and the glucose sensing apparatus in pancreatic islets. Furthermore, the impact of receptor deletion was addressed in both sexes.

## 2. Results

### 2.1. Cannabinoid Receptor Expression in Mouse Pancreatic Islets and Islet Cell Types

**Figure 1:**
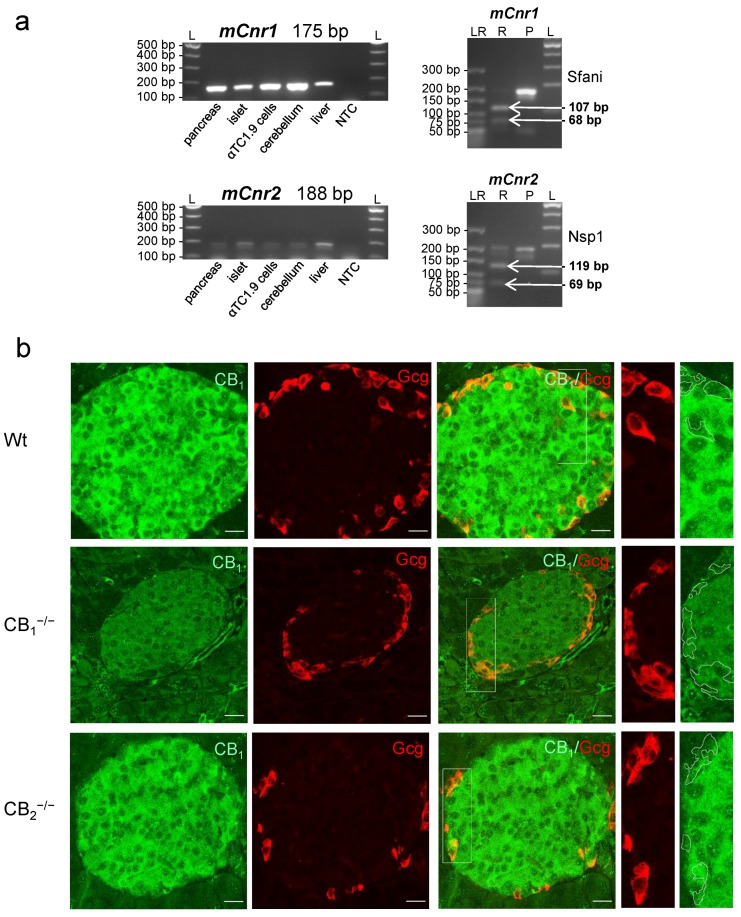
(**a**) Expression of cannabinoid receptor type 1 (*Cnr1*) and 2 (*Cnr2*) mRNA in mouse (m) tissues. RT-PCR followed by gel electrophoresis (left column) revealed the expression of *mCnr1* and *mCnr2* transcripts in different mouse organs, particularly, in pancreatic tissue including the islets of Langerhans and the mouse alpha-cell line αTC1.9. Restriction digestion of the 175 bp *mCnr1* amplicon resulted in defined fragments with molecular sizes of 107 and 68 bp (right column). The restriction analysis of the 188 bp *mCnr2* showed defined fragments with molecular sizes of 119 and 69 bp (right column). NTC: nontemplate control; L: 100 bp ladder; LR: low-molecular-range ladder; P: *Cnr1* or *Cnr2* amplification product; R: restriction fragments. (**b**) Immunohistochemical staining of cannabinoid receptor type 1 (CB_1_) in the pancreatic tissue of wild-type (Wt) mice displayed specific labelling of an islet. Glucagon (Gcg, red) and CB_1_ (green) double-immunolabelling demonstrated the presence of CB_1_, not only in beta-cells, but also in alpha-cells. At higher magnifications of alpha-cells (2 fold, right panels), weaker CB_1_ staining was evident. No immunohistochemical staining of CB_1_ was detected in pancreatic islets of CB_1_^−/−^ knockout mice. In all cases, confocal optical sections were merged and are representative for pancreatic islets of the whole pancreatic tissue from three mice per group. Scale bar 20 µm.

In order to assess the putative effects of cannabinoid receptor knockout, the expression of receptors in a murine alpha-cell line and different organs of wild-type (Wt) mice was verified by RT-PCR. The specificity of primers was checked by restriction analysis, and the sizes of PCR products were confirmed (Figure 1a). Immunolabelling of CB_1_ was evident in pancreatic islets of Wt and CB_2_^−/−^ mice, while only low levels of immunofluorescence were detected in the exocrine tissue. Most of the labelling was found in the centre of the islet, pointing to a higher expression of CB_1_ in pancreatic beta-cells of mouse islets. Double labelling revealed CB_1_ colocalisation in glucagon-producing cells as well. The specificity of the primary antibody was verified earlier [24] and is confirmed in the present study (Figure 1b).

### 2.2. Measurement of Body Weight, Blood Glucose, Plasma Insulin and Glucagon

**Figure 2:**
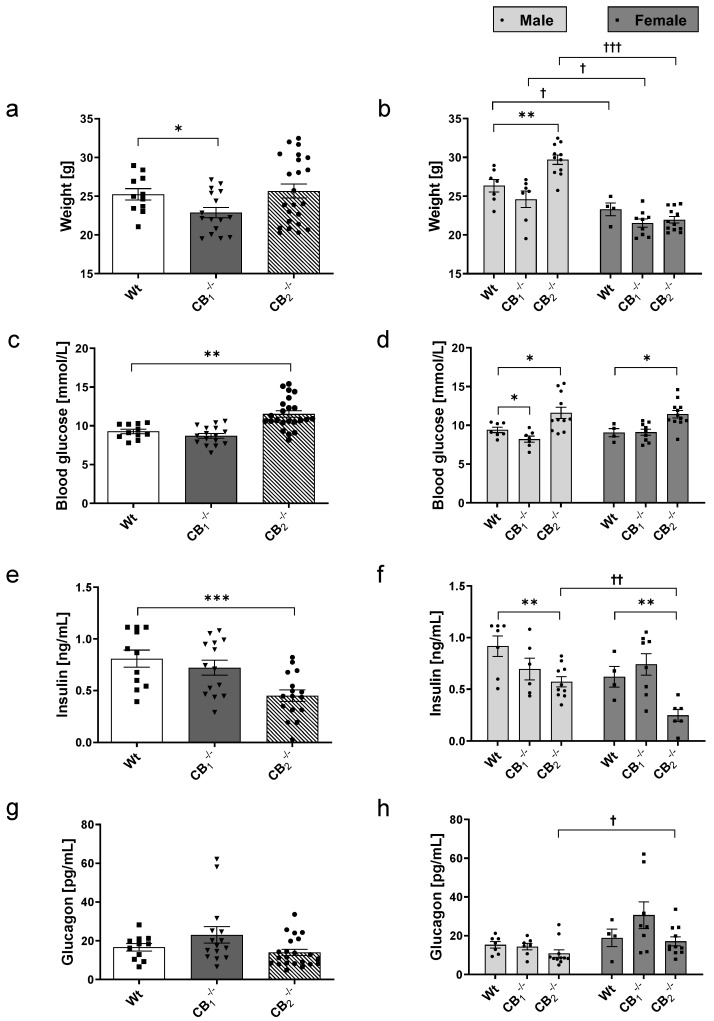
Determination of body weight (**a**,**b**), blood glucose (**c**,**d**), plasma insulin (**e**,**f**) and glucagon (**g**,**h**) of wild-type (Wt) and cannabinoid receptor knockout mouse lines (CB_1_^−/−^, CB_2_^−/−^). (**a**,**b**) CB_1_^−/−^ mice displayed decreased body weights. Male CB_2_^−/−^ mice showed a significantly elevated body weight. Female mice of all groups indicated lower weights then their male counterparts. (**c**,**d**) Male CB_1_^−/−^ mice showed reduced blood glucose values. In contrast, CB_2_^−/−^ mice of both sexes revealed increased blood glucose values. (**e**,**f**) Mean plasma insulin levels were decreased in CB_2_^−/−^ mice of both sexes. In addition, female CB_2_^−/−^ mice showed lower insulin levels than male CB_2_^−/−^ mice. (**g**,**h**) Mean plasma glucagon levels pointed to a slight increase in CB_1_^−/−^ mice. Female CB_1_^−/−^ seemed to be responsible for this increase. Female CB_1_^−/−^ and CB_2_^−/−^ mice showed higher glucagon levels than their respective male counterparts. Values are presented as standard error of the mean (±S.E.M.) with *n* = 11–23 animals per group or *n* = 4–12 animals per group when analysing data for male or female Wt and knockout mice separately. * *p* < 0.05; ** *p* < 0.01; *** *p* < 0.001 for overall group comparisons within male or female Wt and knockout mice; ^†^*p* < 0.05; ^††^ *p* < 0.01; ^†††^ *p* < 0.001 for sex-specific comparisons between male and female Wt or knockout mice; unpaired *t*-test.

Wt mice showed a mean body weight of 25.24 g (Figure 2a). In comparison, CB_1_^−/−^ mice displayed a significant decrease of mean body weight (22.87 g). Without reaching significance, this decline was seen in females (21.53 g; *p* = 0.0981) as well as males CB_1_^−/−^ (24.60 g; *p* = 0.2127), compared to the respective Wt mice (female: 23.29 g; male: 26.35 g). In contrast, the overall body weight of CB_2_^−/−^ mice (25.66 g) was not altered. When analysing data for male and female mice separately, only male CB_2_^−/−^ mice showed increased body weight (29.70 g). In general, the body weight of female mice in all groups was lower than that of male mice (Figure 2b).

Compared to blood glucose levels of Wt mice (Figure 2c), we found no differences in the mean values of peripheral blood glucose in CB_1_^−/−^ mice. However, male CB_1_^−/−^ mice showed statistically significant lower blood glucose levels (CB_1_^−/−^: 8.21 mmol/L; Wt: 9.4 mmol/L). The mean blood glucose level of CB_2_^−/−^ (11.52 mmol/L) was significantly increased with respect to the level of Wt mice (Figure 2c). This increase was seen in both male and female CB_2_^−/−^ (Figure 2d).

Furthermore, CB_1_^−/−^ mice showed similar plasma insulin levels compared to Wt mice (Figure 2e), and no difference was observed when comparing female and male mice (Figure 2f). In contrast, CB_2_^−/−^ mice displayed significantly decreased insulin levels (Wt: 0.81 ng/mL; CB_2_^−/−^: 0.45 ng/mL; Figure 2e). The same finding was evident in a sex-specific analysis. Interestingly, in female Wt mice, slightly lower insulin levels were observed compared to male Wt mice (*p* = 0.0832), which became significant between female and male CB_2_^−/−^ mice (Figure 2f).

The mean plasma glucagon levels were nonsignificantly increased in CB_1_^−/−^ (23.04 pg/mL) compared to Wt mice (16.60 pg/mL, *p* = 0.2305; Figure 2g). Female CB_1_^−/−^ seemed to be responsible for this increase (Figure 2h). In contrast, CB_2_^−/−^ mice (13.96 pg/mL) showed similar glucagon levels to those in Wt mice. Looking at the female and male knockout mice, different glucagon levels were measured. The results displayed higher glucagon levels in female receptor knockout mice (CB_1_^−/−^: 30.61 pg/mL, *p* = 0.0515; CB_2_^−/−^: 17.09 pg/mL) than in male mice from both groups (CB_1_^−/−^: 14.38 pg/mL; CB_2_^−/−^: 10.84 pg/mL; Figure 2h).

Taken together, the elimination of cannabinoid receptor signalling affected body weight, blood glucose as well as plasma insulin and glucagon levels, even though this was differently associated with the type of receptor deleted and the sex. The alterations in blood glucose and plasma insulin levels measured in CB_2_^−/−^ mice were seen in female and male mice, indicating no sex-specific differences.

### 2.3. Measurements of Transcripts and Proteins of Islet Hormones

**Figure 3:**
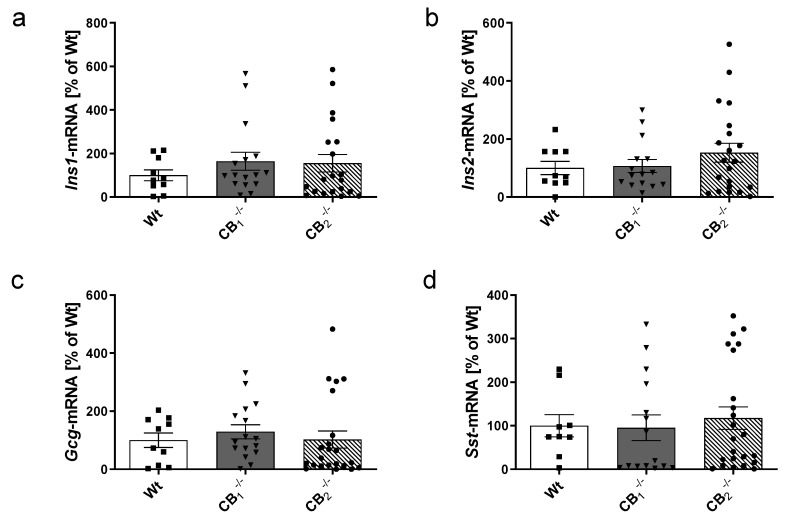
Transcript analysis by real-time RT-PCR of the pancreatic islet hormones (**a**) insulin 1 (*Ins1*), (**b**) insulin 2 (*Ins2*), **(c)** glucagon (*Gcg*), and (**d**) somatostatin (*Sst*) in pancreatic tissue of male and female wild-type (Wt) mice and cannabinoid receptor knockout mouse lines (CB_1_^−/−^, CB_2_^−/−^). No statistically significant differences were measured. The expression level of Wt mice was defined as 100%. Values are presented as mean (±S.E.M.) with *n* = 9–23 mice per group; unpaired *t*-test.

**Figure A1:**
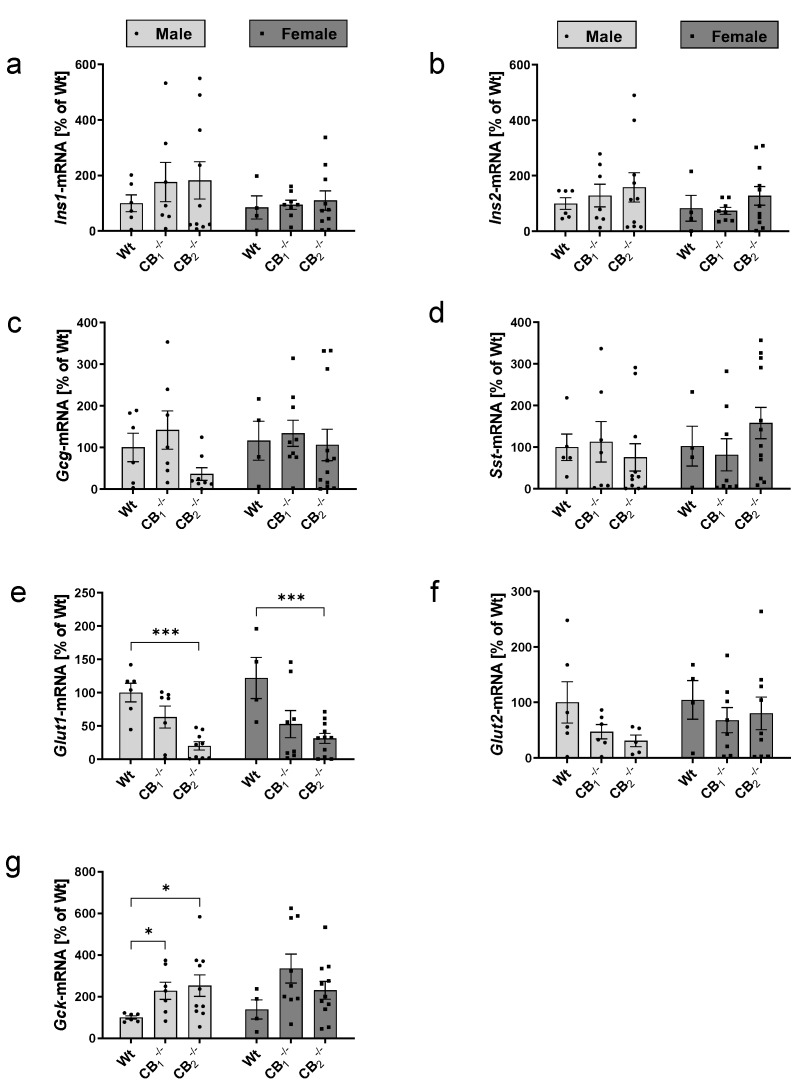
Transcript analysis by real-time RT-PCR of the pancreatic islet hormones (*Ins1*, *Ins2*, *Gcg* and *Sst*), glucose transporters (*Glut1*, *Glut2*) and glucokinase (*Gck*) analysing male and female wild-type (Wt) mice and cannabinoid receptor knockout mouse lines (CB_1_^−/−^, CB_2_^−/−^) separately. No statistical significant differences were measured for *Ins1* (**a**), *Ins2* (**b**), *Gcg* (**c**) and *Sst* (**d**) between male and/or female Wt and knockout mice. (**e**,**f**) Transcript analysis of glucose transporters showed downregulation of *Glut1* as well as *Glut2* for both sexes in knockout mice. (**g**) *Gck* mRNA was upregulated in knockout lines of both sexes. Values are presented as mean (±S.E.M.) with *n* = 4–12 mice per group defining Wt values as 100%. * *p* < 0.05; *** *p* < 0.001 for comparisons between male or female Wt and knockout mice; unpaired *t*-test.

The previous observations regarding the shift of metabolic parameters in cannabinoid receptor knockout mice (Figure 2) led us to investigate the effects of cannabinoid receptor knockout on the function of pancreatic islets. To this end, we determined the transcript levels of the pancreatic islet hormones insulin (*Ins1* and *Ins2*), glucagon (*Gcg*) and somatostatin (*Sst*) in pancreatic tissue (Figure 3). The specificity of PCR products of pancreatic hormones was confirmed in an earlier study [25]. When comparing islet hormone transcript levels between Wt and knockout mice, no statistically significant differences were observed (Figure 3a–d). In addition, there were no significant variations considering sex-specific analyses (Figure A1a–d).

**Figure 4:**
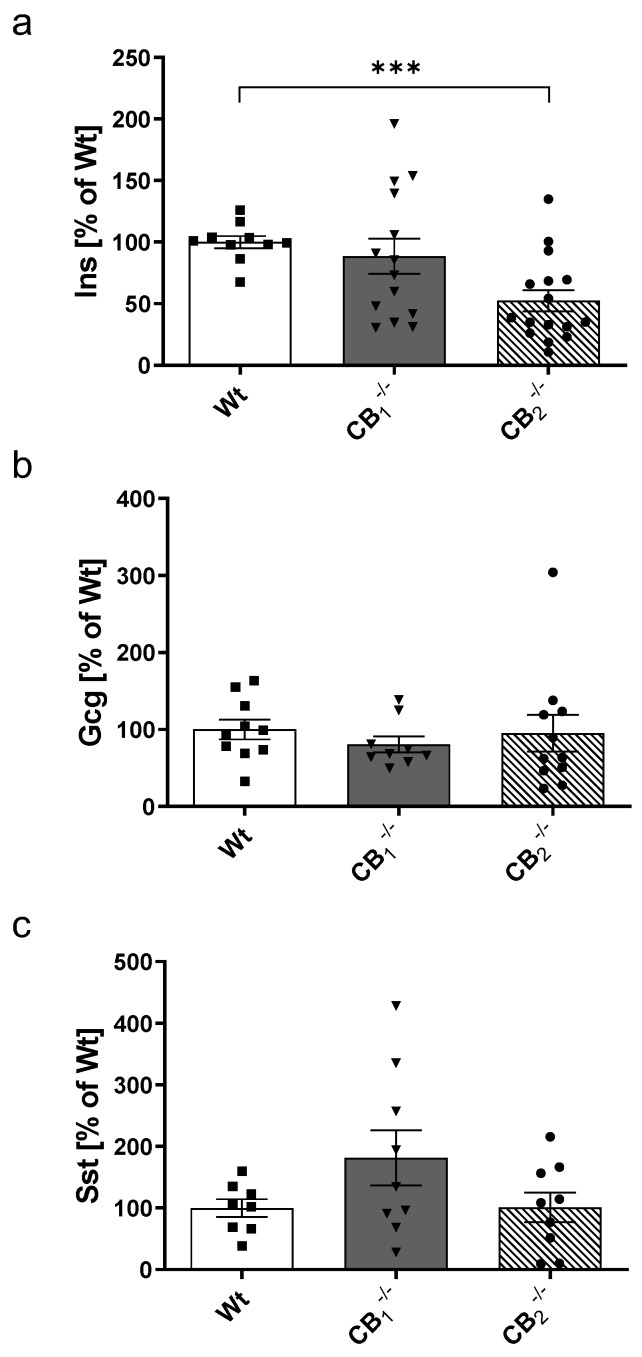
Western blot analysis of insulin (**a**), glucagon (**b**) and somatostatin (**c**) protein in pancreatic tissue of male and female wild-type (Wt) mice and cannabinoid receptor knockout mouse lines (CB_1_^−/−^, CB_2_^−/−^). (**a**) Insulin protein was decreased in the pancreatic tissue of CB_2_^−/−^ mice. (**b**) Glucagon and (**c**) Somatostatin protein content displayed no significant differences, although a nonsignificant increase in CB_1_^−/−^ was observed. Values are presented as mean (±S.E.M.) with *n* = 8–16 mice per group. Each protein level of male and female Wt mice was defined as 100%. *** *p* < 0.001; unpaired *t*-test.

**Figure A2:**
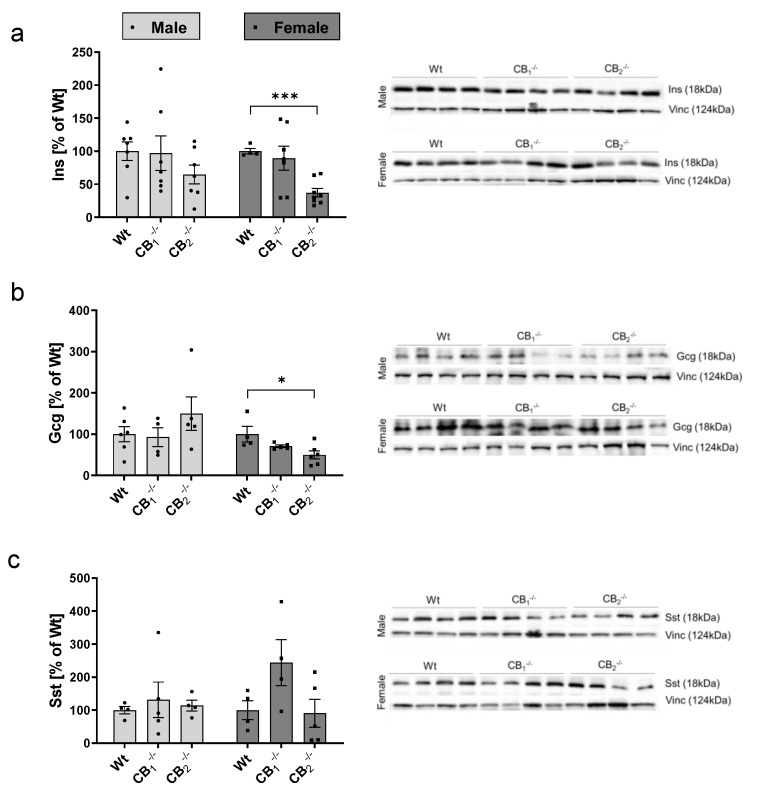
Western blot analysis of pancreatic islet hormones analysing male and female wild-type (Wt) mice and cannabinoid receptor knockout mouse lines (CB_1_^−/−^, CB_2_^−/−^) separately. (**a**) A decrease in insulin (Ins) protein was found in male and female CB_2_^−/−^. (**b**) Glucagon (Gcg) protein was decreased in female CB_2_^−/−^ mice. (**c**) Somatostatin (Sst) protein seemed to be increased in female CB_1_^−/−^ mice. Analysis of male and female mice was done separately on different immunoblots, defining male and female Wt protein levels as 100%. A representative western blot of each protein is shown in the right column showing specific single bands as indicated. Vinculin (Vinc) was used as control protein. Values are presented as mean (±S.E.M.) with *n* = 4–9 mice per group. * *p* < 0.05; *** *p* < 0.001 for comparisons within male or female Wt and knockout mice; unpaired *t*-test.

Western blot analysis of insulin showed no change in protein content in CB_1_^−/−^, but a significant reduction of 48% in CB_2_^−/−^ mice (Figure 4a). Considering sexes, a decrease of insulin was evident in both groups, with a significant decrease displayed in female CB_2_^−/−^ mice (Figure A2a). In contrast, total glucagon protein levels were not altered in CB_1_^−/−^ and CB_2_^−/−^ (Figure 4b). But in male CB_2_^−/−^ mice, an increased glucagon level was measured when compared to the Wt, whereas in female knockout mice, a decrease was evident (Figure A2b). Overall, higher somatostatin protein amounts were detected without reaching the level of significance in CB_1_^−/−^ mice (Figure 4c). Interestingly, this trend was seen in female CB_1_^−/−^ mice only (Figure A2c).

### 2.4. Changes in Key Components of the Glucose Sensing Machinery

To provide possible explanations for changes in blood glucose levels in cannabinoid receptor knockout mice, we investigated the expression of glucose transporter Glut1 (encoded by *Slc2a1*) and Glut2 (encoded by *Slc2a2*), as well as the glucose sensor glucokinase (Gck) in pancreatic tissue.

**Figure 5:**
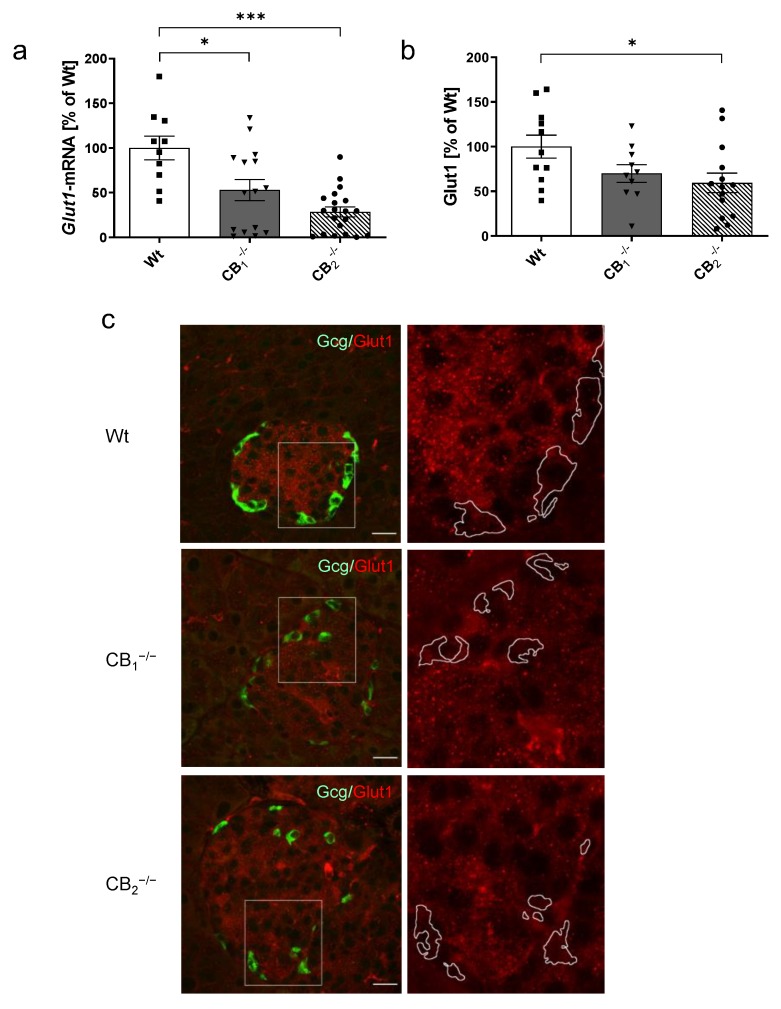
Analysis of glucose transporter 1 mRNA levels (*Glut1*) and protein (Glut1) in pancreatic tissue of male and female wild-type (Wt) mice and cannabinoid receptor knockout mouse lines (CB_1_^−/−^, CB_2_^−/−^). (**a**) Relative mRNA expression level of *Glut1* was decreased in both knockout mouse lines (*n* = 10–20). (**b**) In addition, protein content of Glut1 was also decreased in CB_1_^−/−^ and CB_2_^−/−^ mice (*n* = 10–14). (**c**) Immunohistochemical staining of Glut1 (red) and colabelling of glucagon (Gcg, green) confirmed the localisation of Glut1 in both alpha- and beta-cells. A magnification of each islet (2.5 fold, box) is shown on the right column, and revealed the reduction of Glut1 in knockout mouse lines. In all cases, confocal optical sections were merged and are representative for pancreatic islets analysed in the whole pancreatic tissue from three mice per group. Values are presented as mean (±S.E.M.) and Wt values were defined as 100%. * *p* < 0.05; *** *p* < 0.001; unpaired *t*-test. Scale bar 20 µm.

**Figure A3:**
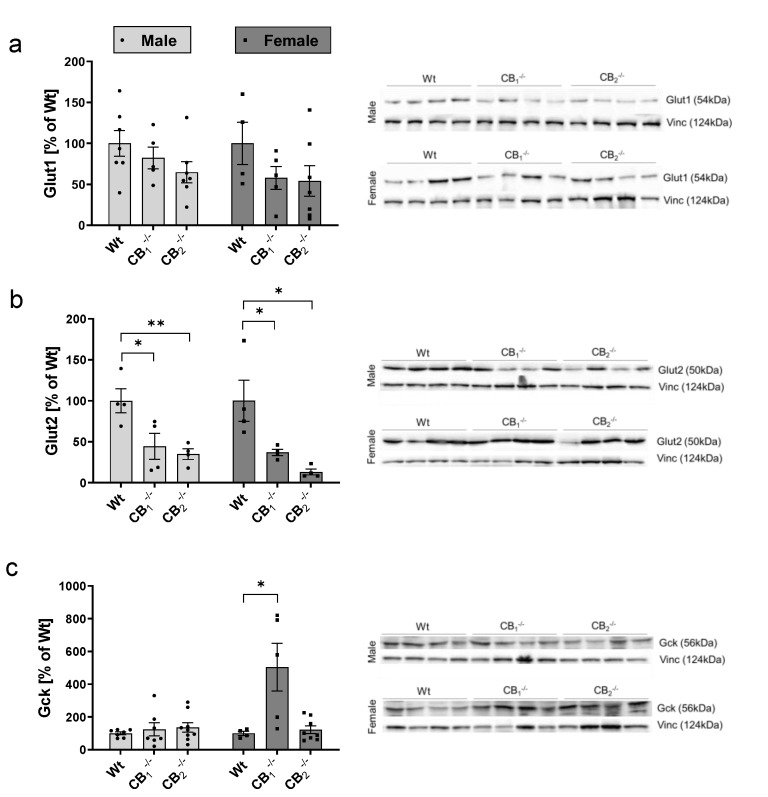
Western blot analysis of glucose transporters (Glut1, Glut2) and glucokinase (Gck) in pancreatic tissue analysing male and female wild-type (Wt) mice and cannabinoid receptor knockout mouse lines (CB_1_^−/−^, CB_2_^−/−^) separately. (**a**) Glut1 seemed to be decreased in CB_1_^−/−^ and CB_2_^−/−^ knockout mice of both sexes. (**b**) Glut2 protein was significantly decreased in CB_1_^−/−^ and CB_2_^−/−^ knockout mice of both sexes. (**c**) Gck protein level was increased in female CB_1_^−/−^ mice. Analysis of male and female mice was done separately on different immunoblots, defining male and female Wt protein levels as 100%. A representative western blot of each protein is shown in the right column showing specific single bands as indicated. Vinculin (Vinc) was used as control protein. Values are presented as mean (±S.E.M.) with *n* = 4–9 mice per group. * *p* < 0.05; ** *p* < 0.01 for comparisons within male or female Wt and knockout mice; unpaired *t*-test.

Compared to Wt, the gene expression of *Glut1* was significantly downregulated in CB_1_^−/−^ and CB_2_^−/−^ mice (a decrease of 47% in CB_1_^−/−^ and of 71% in CB_2_^−/−^, Figure 5a). The same pattern was reflected in female and male groups (Figure A1e). In accordance, protein levels were lower in both knockout mice, but showed significant changes in CB_2_^−/−^ only (a reduction of 30% in CB_1_^−/−^, *p* = 0.0853 and of 41% in CB_2_^−/−^, Figure 5b). Nonsignificant decreases were observed in male and female groups (Figure A3a). In immunohistochemistry, Glut1 was presented in the pancreatic islet with enhanced immunolabelling in the centre of the islet mainly occupied by beta-cells. Colabelling of Glut1 with glucagon revealed a colocalisation of Glut1 in alpha-cells as well, albeit with weaker staining (Figure 5c). The reduced immunolabelling of Glut1 was evident in both knockout mice.

**Figure 6:**
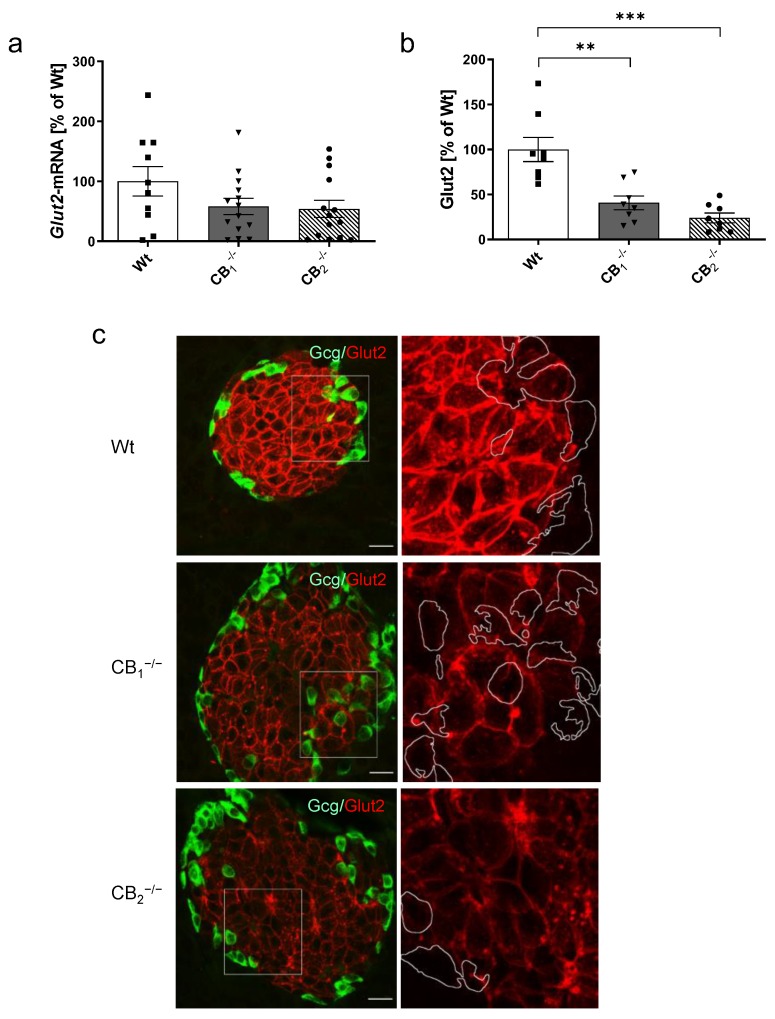
Analysis of glucose transporter 2 mRNA (*Glut2*) and protein (Glut2) in pancreatic tissue of male and female wild-type (Wt) mice and cannabinoid receptor knockout mouse lines (CB_1_^−/−^, CB_2_^−/−^). (**a**) Relative mRNA expression level of *Glut2* was decreased in both knockout mouse lines (*n* = 10–14). (**b**) In addition, protein content of Glut2 was also decreased in CB_1_^−/−^ and CB_2_^−/−^ mice (*n* = 8). (**c**) Immunohistochemical staining of Glut2 (red) and colabelling of glucagon (Gcg, green) was shown to be prominent in the pancreatic islet displaying a typical membrane-concentrated distribution pattern in beta-cells. As shown in the overview (left column), as well as in the magnification of each islet (2.5 fold, box) on the right column, knockout mice yielded weaker Glut2 immunostaining of the membranes in beta-cells, but also increased cytoplasmic accumulations, showing stronger accentuation in CB_2_^−/−^ mice. In all cases, confocal optical sections were merged and are representative for pancreatic islets analysed in the whole pancreatic tissue from three mice per group. Values are presented as mean (±S.E.M.) and Wt values were defined as 100%. ** *p* < 0.01; *** *p* < 0.001; unpaired *t*-test. Scale bar 20 µm.

*Glut2* transcript levels were, although not statistically significant, decreased in knockout mice (Figure 6a). A nonsignificant reduction of *Glut2* transcripts was seen in both male and female mice (Figure A1f). Considering Glut2 protein, a significant decrease was measured for CB_1_^−/−^ and CB_2_^−/−^ mice (Figure 6b). An overall significant decrease of protein was seen in both sexes (Figure A3b). Immunohistochemistry revealed the labelling of Glut2 to be predominant in pancreatic islets and, more specifically, in pancreatic beta-cells restricted to the cell membrane (Figure 6c). Knockout mice yielded weaker immunostaining of the membranes, but increased cytoplasmic labelling, as seen by small dots in the cytoplasm. In CB_2_^−/−^, this altered pattern was more accentuated, showing more accumulations in the cytoplasm and a modified appearance of the membrane structure.

**Figure 7:**
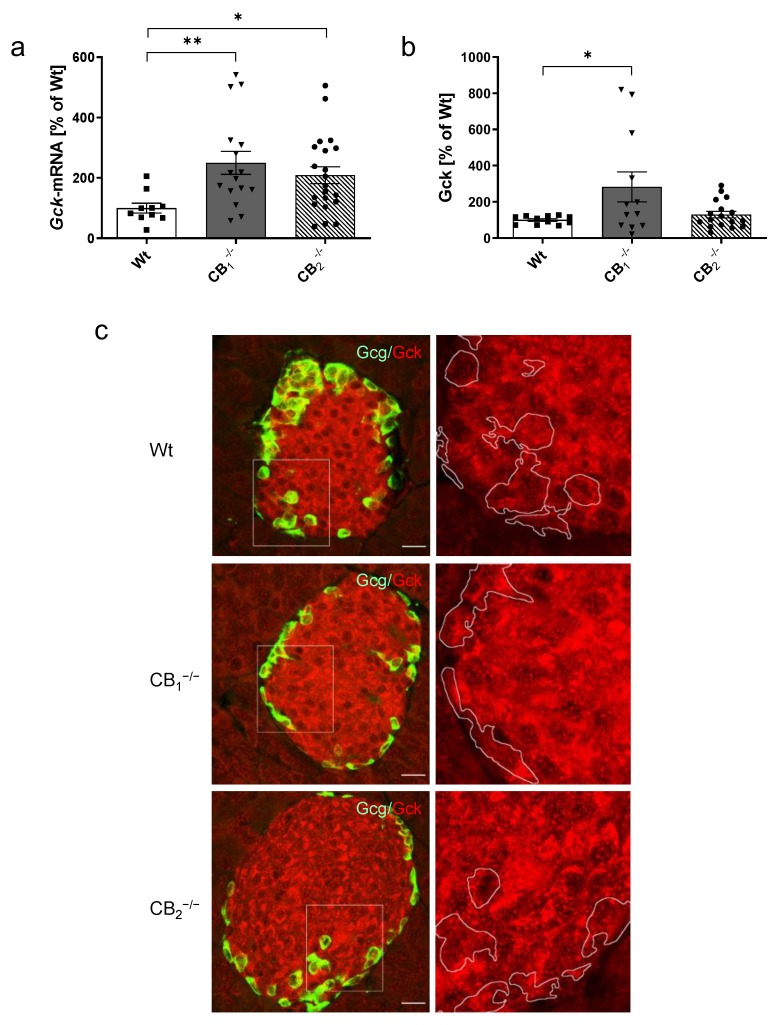
Analysis of glucokinase mRNA (*Gck*) and protein (Gck) in pancreatic tissue of male and female wild-type (Wt) mice and cannabinoid receptor knockout mouse lines (CB_1_^−/−^, CB_2_^−/−^). (**a**) The relative mRNA expression level of *Gck* was increased in both knockout mouse lines compared to Wt mice (*n* = 10–21). (**b**) An increase in pancreatic protein content of Gck was seen only in CB_1_^−/−^ mice (*n* = 11–17). (**c**) Immunohistochemical staining of Gck (red) and colabelling of glucagon (Gcg, green) demonstrated labelling of Gck in alpha- and beta-cells with a heterogeneous cytoplasmic labelling surrounding the nuclei in a ring-like manner. As indicated at higher magnification (2.5 fold, right column) of each islet (box), CB_1_^−/−^ mice also showed a heterogeneous Gck distribution pattern with accumulations around the nuclei, with high and low density distribution in the cytoplasmic region. In CB_2_^−/−^, the heterogeneous detection of Gck was more prominent, displaying distinct accumulations in the perinuclear region with semilunar appearance. In all cases, confocal optical sections were merged and are representative for pancreatic islets of whole pancreatic tissue from three mice per group. Values are presented as mean (±S.E.M.) and Wt values were defined as 100%. * *p* < 0.05; ** *p* < 0.01; unpaired *t*-test. Scale bar 20 µm.

*Gck* transcript levels were significantly increased in both CB_1_^−/−^ and CB_2_^−/−^ compared to Wt mice (Figure 7a). This increase seemed to be sex-independent (Figure A1g). However, protein analysis revealed a significant increase of Gck protein in CB_1_^−/−^ only (Figure 7b), which was restricted to female CB_1_^−/−^ mice (Figure A3c). Gck immunolabelling in pancreatic islets of Wt mice displayed a heterogeneous cytoplasmic staining, where it appears to surround the nuclei in a ring-like manner (Figure 7c). CB_1_^−/−^ displayed a heterogeneous distribution pattern of Gck labelling with accumulations around the nuclei with areas of high and low density in the cytoplasmic region. In CB_2_^−/−^, the heterogeneous staining of Gck was even more prominent and seemed to be reinforced by distinct accumulations in the perinuclear region, displaying a semilunar appearance (Figure 7c).

## 3. Discussion

The present work focuses on the impact of deletion of cannabinoid receptor types CB_1_ or CB_2_ on pancreatic islet hormone secretion and glucose metabolism. Until now, the expression and distribution of both cannabinoid receptors in pancreas was controversial in mice, showing the presence of CB_1_ mainly on nonbeta-cells including alpha- or delta-cells [26,27,28]. However, CB_1_ and CB_2_ receptors were also found in islet beta-cells [10,11]. Another group confirmed the presence of CB_1_ but neglected that of CB_2_ in mouse islets [29]. In accordance with studies of Li and colleagues [10,11], we detected mRNA of both CB_1_ and CB_2_ in mouse pancreatic islets. CB_1_ immunolabelling was restricted to pancreatic islets, displaying a stronger signal in beta-cells and a weaker signal in alpha-cells.

Knockout of CB_1_ led to decreased body weight in mice without any notable impact on islet hormones in plasma and pancreas. Similar to our findings, lower body weights were reported in CB_1_^–/–^ mice compared to CB_1_*^+/+^* mice, whereas plasma insulin and glucose levels were not different between strains [30]. In contrast, another study described an upregulation of insulin gene expression in CB_1_^–/–^ mice and an increased level of pro-insulin compared to those of age-matched CB_1_^+/+^ mice [21]. As shown in rodent models in general, pharmacological inhibition of the CB_1_ downregulated food intake and body weight [31]. In line with these findings, anti-obesity effects were reported as a result of chronic CB_1_ receptor antagonist treatment [32] or for whole-body CB_1_ knockout mice [15,30]. 

Knockout of CB_1_ led to decreased body weight in mice without any notable impact on islet hormones in plasma and pancreas. Similar to our findings, lower body weights were reported in CB_1_^−/−^ mice compared to CB_1_*^+/+^* mice, whereas plasma insulin and glucose levels were not different between strains [30]. In contrast, another study described an upregulation of insulin gene expression in CB_1_^−/−^ mice and an increased level of pro-insulin compared to those of age-matched CB_1_^+/+^ mice [21]. As shown in rodent models in general, pharmacological inhibition of the CB_1_ downregulated food intake and body weight [31]. In line with these findings, anti-obesity effects were reported as a result of chronic CB_1_ receptor antagonist treatment [32] or for whole-body CB_1_ knockout mice [15,30]. 

Interestingly, the generation of a cell-specific male CB_1_R knockout mouse model (beta-CB_1_R^−/−^) allowed us to determine the role of CB_1_ specifically in beta-cells of CB_1_^−/−^ mice [6]. Contrary to our findings, beta-CB_1_R^−/−^ mice showed no changes of body weight and a significant increase of fasting plasma insulin levels. Similar to the previous study [6], a lowered fasting blood glucose level was also observed in the male CB_1_^−/−^ mice in our study. In view of these findings, our knockout data must be considered in the context of a whole-body metabolism, taking into consideration the well-established interplay between pancreas and liver [33]. In this context, the liver is known to express CB_1_ and CB_2_ receptors [34].

The CB_2_^−/−^ mice showed decreased plasma insulin levels combined with increased blood glucose levels in both sexes, and increased body weights in male mice. Another study reported in two-month-old male CB_2_^−/−^ mice similar body weight values to those of normoglycaemic, age-matched, wild-type mice [18]. Increased body weight of CB_2_^−/−^ mice was measured in other studies [19,35], which is in accordance with our data considering male CB_2_^−/−^ mice only. 

Sex-specific differences were measured for serum glucagon levels in our study, displaying higher levels in female CB_1_^−/−^ mice and, in general, higher levels in female knockout mice compared to male knockout mice. This strongly supports a sex-specific impact of receptor loss, which has not been considered so far, since most studies used male rodents. However, there is an increasing number of studies suggesting a sexually dimorphic function of the CB_1_ receptor [36], indicating sex-specific differences within the cannabinoid-regulated biology [37]. Beyond sex-specific characteristics in cannabinoid receptor pharmacology, there is growing preclinical evidence for an impact of gonadal hormones, particularly estradiol, on cannabinoid receptor density or function [38], providing a possible explanation for our sex-specific findings. 

In feeding mouse models, CB_2_ receptor deletion was associated with impaired glucose clearance [19], supporting our findings of elevated blood glucose and hormonal differences in CB_2_^−/−^. Using diet-induced obesity mice, CB_2_ receptor agonists were shown to be efficacious in reducing body weight and obesity-associated metabolic parameter, e.g., insulin. Furthermore, acute administration of CB_2_-agonist JWH-015 produced a significant improvement in glucose clearance [39]. 

We propose that the deletion of CB_1_ or CB_2_ in our study resulted in an impairment of glucose metabolism, since key components of the glucose sensing machinery, such as glucose transporters and glucokinase, were affected in both knockout mouse lines. Sand rats represent a well-established model of nutritionally-induced, noninsulin-dependent, type 2 diabetes. The loss of immunostaining for the Glut2 glucose transporter in the plasma membrane of the pancreatic beta-cells became evident when these animals subsequently developed hyperglycaemia [40]. Furthermore, in type 2 diabetic Goto-Kakizaki rats, which were characterised by changes in blood glucose and insulin, Glut2 was reduced, and changes in its distribution patterns were evident [41]. Accordingly, glucose transporter 2 was reduced in both knockout mouse lines in the present study, although in CB_2_^−/−^ the reduction seemed to be reinforced by cytoplasmic accumulations of Glut2 immunolabelling, indicating a reduced number of membrane-bound functional transporters. In diabetic rodents, impaired glucose-stimulated insulin secretion was associated with a markedly reduced expression of Glut2, regulated on both the mRNA and protein levels [23], thereby supporting the well-accepted fact that Glut2 is required in order to maintain normal glucose homeostasis and normal endocrine pancreas function. Notably, in renal tissue, the Glut2 expression and translocation changes are under the regulation of the CB_1_ receptor [42]. Thus, CB_1_ affected its dynamic membrane translocation and modulated glucose reabsorption. High glucose and ACEA (a CB_1_ agonist) treatment caused an increase of perinuclear translocation of Glut2 in renal MDCK II cells, whereas under CB_1_ antagonism with JD5037, a decrease of perinuclear Glut2 was demonstrated. Inhibition of CB_1_ also downregulated the Glut2 expression in renal cells [42]. The aforementioned processes could play a role in our CB_1_^−/−^ and perhaps CB_2_^−/−^ mice with lower levels of glucose transporters incorporated into the cell membrane. Thus, we hypothesise that the genetic knockout of CB_1_, but also CB_2_, has the potential to influence the trafficking of glucose in beta-cells by affecting the glucose transporters Glut1, and especially Glut2 at transcriptional and protein levels, as well as their translocation. Glut1 is a high affinity and low K_m_ transporter responsible for glucose influx into beta-cells which becomes activated at low glucose concentrations, allowing rapid equilibration to occur of extra- and intra-cellular glucose. Glut1 was also affected after CB_1_ or CB_2_ loss. In this context, a direct functional link between the transporters responsible for glucose uptake and the capacity of beta-cells to increase insulin secretion was reported [43]. However, the detailed underlying mechanisms in association with cannabinoid receptors have been investigated neither in rodent nor in human beta-cells. CB_1_ was shown to regulate the Glut2 expression, its membrane translocation and activity by a signalling mechanism that involves elevating cytosolic Ca^2+^ levels and activating the upstream modulator of Glut2, protein kinase C-β1 [42,44].

Glucokinase plays a critical role in glucose homeostasis, and is described as a candidate diabetes mellitus gene [45,46]. In type 2 diabetic rodent models, a decrease of pancreatic glucokinase expression was paralleled by a reduced staining in the pancreatic islet [40,41], which is contrary to our observations. Glucose functions as a modulator of the pancreatic glucokinase, directly affecting levels in beta-cells, and stimulates insulin secretion [45]. Thus, the hyperglycaemia that occurs in CB_2_^−/−^ mice may be responsible for the higher level of glucokinase in the pancreatic islets, which might be a way to compensate for lower plasma insulin levels. Nonetheless, this does not give a plausible explanation for changes seen in CB_1_^−/−^ mice, as their blood glucose levels were comparable to those of Wt mice. So besides glucose, insulin regulates beta-cell glucokinase expression [47]. In accordance with this, a recent study investigated an interaction between the CB_1_ and the insulin receptor, and showed an increased expression of glucokinase in mouse beta-cells after silencing the CB_1_ receptor, an effect which was lost when the insulin receptor was missing. Furthermore, CB_1_^−/−^ islets in pancreata from overnight-fasted CB_1_^−/−^ mice displayed increased glucokinase expression compared to islets from CB_1_^+/+^ mice [21].

In summary, the present study underscores the importance of CB_1_ and CB_2_ signalling for pancreatic islet cell function with underlying different roles of CB_1_ and CB_2_, which is supported by [48]. CB_1_ or CB_2_ receptor knockout resulted in alterations in the glucose sensing recognition apparatus, including glucose transporters and glucokinase, which could explain metabolic changes. In beta-cells, glucokinase is the first enzyme phosphorylating glucose, and is thus the primary determinant for the glycolytic flux rate [22]. Surprisingly, our CB_2_^−/−^ mice revealed the most alterations, although it is known that CB_2_ is expressed to a much lower extent (100-fold difference) than CB_1_ [6,8,27,49]. Notably, the altered glucose sensing in pancreatic beta-cells, combined with lower insulin levels and higher blood glucose in CB_2_^−/−^ mice, should be taken into consideration in view of the development of pharmacological cannabinoid receptor antagonists in diabetes therapy. Otherwise, it should be remembered that only one of the two receptors in our mice was deleted, while the other was functionally active. Hence, we cannot exclude a compensatory mechanism of the other cannabinoid receptor type. So, further studies will be needed to clarify different roles of CB_1_ and CB_2_ signalling and their mechanism in pancreatic islet cells.

## 4. Materials and Methods 

### 4.1. Animals and Tissue Sampling

All animal experiments were performed in accordance with the Policy on Ethics and the Policy on the Use of Animals in Neuroscience Research as indicated in the directive 2010/63/EU of the European Parliament and of the Council of the European Union on the protection of animals used for scientific purposes and were approved by the local authorities for care and use of laboratory animals (State of Saxony-Anhalt, Germany; number I11M27). Criteria according to the 3Rs (Replacement, Reduction and Refinement) were considered. Male and female mice of global cannabinoid receptor knockout lines, representing the CB_1_ (CB_1_^−/−^; *n* = 16: 9 female, 7 male), CB_2_ receptor knockout (CB_2_^−/−^; *n* = 23: 12 female, 11 male) mice and their wild-type littermates (CB_1_^+/+^, Wt; *n* = 11: 4 female, 7 male) were bred as previously described [12,14]. The mice, taken from our own breeding colony, had a C57BL/6N background. All animals were housed in a temperature- and humidity-controlled vivarium with a 12-hr light-dark cycle (L:D = 12:12, light on: 06:00 a. m.), and had access to food and water ad libitum, feeding on a standard diet.

Mice at 10–12 weeks of age were used. Food was taken away 3 h before mice were killed under deep anaesthesia during the light period (10:00–14:00 a.m.). Body weight was determined and blood glucose levels from tail-tip samples were analysed with a measuring device (MediSense Precision, Wiesbaden, Germany). Blood samples were acquired by heart ventricle puncture. After centrifugation, the supernatant was stored at −80 °C. Pieces of organs were stored at −80 °C. For gene expression analysis, pieces of pancreata were immediately preserved in RNAlater (Ambion Inc., Austin, TX, USA). Stored probes of alphaTC1.9 cells were used for RT-PCR.

Isolated islets were prepared for qualitative mRNA analysis. Briefly, the mouse was killed by cervical dislocation, and the pancreas was perfused by injection of 3 mL of Collagenase-P (1 mg/mL; Roche, Mannheim, Germany) in Hank’s buffered salt solution (HBSS) containing 25 mM HEPES and 0.5% (w/v) BSA into the common bile duct. Subsequently, the perfused pancreas was digested in 2 mL of collagenase solution for 9–10 min at 37 °C. With the help of a cannula (18G × 11′2), islets were mechanically detached from exocrine parts and washed for several times with HBSS. Finally, islets were purified by hand picking in RPMI 1640 supplemented with 10% FCS, 100 U/mL penicillin and 100 µg/mL streptomycin. Total islet RNA was extracted with TRIzol.

### 4.2. RNA Extraction, DNAse Treatment and Real-Time RT-PCR

Total RNA was isolated using a standard protocol for TRIzol extraction (TRI Reagent^®^, Sigma-Aldrich GmbH, Taufkirchen, Germany). DNAse treatment (DNA- free™; Ambion Inc. Austin, TX, USA), and reverse transcription (Promega Inc., Madison, WI, USA) was carried out as indicated according to the manufacture’s protocols. 

Real-time RT-PCR was carried out using 7900HT Fast Real-Time PCR system (Applied Biosystems, via Thermo Fisher Scientific, Karlsruhe, Germany). Primer sequences are listed in Supplementary Table S1. For quantification of the relative expression levels of target genes, the ΔΔ-C_t_ method [50] was used. The expression of beta-actin (*Actb*) was used to normalise the target genes. The identity of amplicons was verified by restriction analysis and agarose gel electrophoresis.

### 4.3. Western Blot

All mouse pancreatic probes were lysed with RIPA-buffer. Western blot analysis was done using a standard protocol. Proteins (25 µg) were blotted on a nitrocellulose membrane and detected using Luminata™ Forte Western HRP Substrate (Millipore Corporation, Billerica, USA) in a Fusion-FX-7 imager and quantified with the Bio1D-software (PEQLAB, Erlangen, Germany). The following antibodies were used: anti-vinculin (monoclonal rabbit, EPR8185; Abcam, Cambridge, UK), anti-insulin (polyclonal rabbit, STJ24210; St John‘s Laboratory, London, United Kingdom), anti-glucagon (polyclonal rabbit, PA5-13442; Thermo Fisher, Waltham, MA, USA), anti-somatostatin (polyclonal rabbit, STJ95730; St John’s Laboratory). Antibodies for glucose transporters and glucokinase were the same as those applied for immunohistochemistry (Suppl. Table S2).

### 4.4. Immunohistochemistry

From fixed whole pancreata (4% Roti-Histofix; Carl Roth GmbH, Karlsruhe, Germany), sections of 5 µm thickness were deparaffinised and heated under standardised conditions (120 °C for 3 min) in 0.05 mol/L Tris-buffered saline (pH 9.5) in a Pascal pressure chamber (S2800, DakoCytomation, Carpinteria, CA, USA). For blocking, sections were incubated in phosphate-buffered saline (PBS, pH 7.4) containing 5% normal goat serum (Dianova GmbH, Hamburg, Germany) and 0.3% Triton X-100 (Sigma-Aldrich GmbH, Taufkirchen, Germany) for 1 h. An overnight incubation with primary antibodies was done at 4 °C (Suppl. Table S2). Afterwards, sections were treated with secondary antibodies for 1 h at room temperature (Suppl. Table S2). Control sections were processed with normal goat serum, substituting the primary antibodies to check for unspecific bindings of secondary antibodies.

### 4.5. Confocal laser Scanning Microscopy

Fluorescence-labelled pancreatic tissues from three Wt, CB_1_^−/−^ and CB_2_^−/−^ mice of both sexes were analysed by confocal laser scanning microscopy (Leica TCS SPE, Wetzlar, Germany). Viewing the whole tissue, 6–17 islets per mouse were randomly scanned. Images consisting of 2048 × 2048 pixel were recorded using a 40× oil-immersion objective and a zoom factor of 1.5. CLSM images consisted of stacks of three consecutive virtual sections which were combined in a single image using the maximum projection of the image analysis software Fiji. Borders of glucagon containing alpha-cells were determined by using an Auto Local Threshold and a Gaussian Blur filter. By an automated operator, the staining was detected in the converted binary image and defined as region of interest (ROI).

### 4.6. Measurements of Plasma Insulin and Glucagon

Insulin and glucagon concentrations were measured in mouse plasma using a Mouse Ultrasensitive Insulin ELISA (ALPCO, Salem, NH, USA) and a Mouse Glucagon ELISA (Crystal Chem, Zaandam, Netherlands) according to the manufacturer’s instructions.

### 4.7. Statistical Analysis

For statistical analyses, CB_1_^−/−^ or CB_2_^−/−^ mice were compared to Wt mice (including sex differences) using an unpaired *t*-test (Prism 8, GraphPad Software Inc., San Diego, CA, USA). Unpaired *t*-test was chosen to compare the mean values obtained in Wt and knockout mice, as they showed differences in one factor. Groups were considered to be significantly different at *p* < 0.05 (95% confidence interval). Data are presented as mean standard error of the mean (± S.E.M).

## Supplementary Materials

Supplementary materials can be found at https://www.mdpi.com/1422-0067/21/9/3168/s1. Table S1: Primers and sequences; Table S2: Primary and secondary antibodies for immunohistochemistry.

## Abbreviations

CB_1_	cannabinoid receptor type 1
CB_1_^−/−^	CB_1_-knockout mice
CB_1_^+/+^	wild-type littermate
CB_2_	cannabinoid receptor type 2
CB_2_^−/−^	CB_2_-knockout mice
ECS	endocannabinoid system
GPCR	G-protein coupled receptor
Wt	wild-type

## References

[1] Amisten S., Salehi A., Rorsman P., Jones P.M., Persaud S.J. (2013). An atlas and functional analysis of G-protein coupled receptors in human islets of Langerhans. Pharmacol. Ther..

[2] Reimann F., Gribble F.M. (2016). G protein-coupled receptors as new therapeutic targets for type 2 diabetes. Diabetologia.

[3] Munro S., Thomas K.L., Abu-Shaar M. (1993). Molecular characterization of a peripheral receptor for cannabinoids. Nature.

[4] Matsuda L.A., Lolait S.J., Brownstein M.J., Young A.C., Bonner T.I. (1990). Structure of a cannabinoid receptor and functional expression of the cloned cDNA. Nature.

[5] Gruden G., Barutta F., Kunos G., Pacher P. (2016). Role of the endocannabinoid system in diabetes and diabetic complications. Br. J. Pharmacol..

[6] González-Mariscal I., Montoro R.A., Doyle M.E., Liu Q.-R., Rouse M., O’Connell J.F., Santa-Cruz Calvo S., Krzysik-Walker S.M., Ghosh S., Carlson O.D. (2018). Absence of cannabinoid 1 receptor in beta cells protects against high-fat/high-sugar diet-induced beta cell dysfunction and inflammation in murine islets. Diabetologia.

[7] Jourdan T., Godlewski G., Kunos G. (2016). Endocannabinoid regulation of β-cell functions: Implications for glycaemic control and diabetes. Diabetes Obes. Metab..

[8] Bermúdez-Silva F.J., Suárez J., Baixeras E., Cobo N., Bautista D., Cuesta-Muñoz A.L., Fuentes E., Juan-Pico P., Castro M.J., Milman G. (2008). Presence of functional cannabinoid receptors in human endocrine pancreas. Diabetologia.

[9] Matias I., Gonthier M.-P., Orlando P., Martiadis V., de Petrocellis L., Cervino C., Petrosino S., Hoareau L., Festy F., Pasquali R. (2006). Regulation, function, and dysregulation of endocannabinoids in models of adipose and beta-pancreatic cells and in obesity and hyperglycemia. J. Clin. Endocrinol. Metab..

[10] Li C., Jones P.M., Persaud S.J. (2011). Role of the endocannabinoid system in food intake, energy homeostasis and regulation of the endocrine pancreas. Pharmacol. Ther..

[11] Li C., Bowe J.E., Jones P.M., Persaud S.J. (2010). Expression and function of cannabinoid receptors in mouse islets. Islets.

[12] Marsicano G., Wotjak C.T., Azad S.C., Bisogno T., Rammes G., Cascio M.G., Hermann H., Tang J., Hofmann C., Zieglgänsberger W. (2002). The endogenous cannabinoid system controls extinction of aversive memories. Nature.

[13] Zimmer A., Zimmer A.M., Hohmann A.G., Herkenham M., Bonner T.I. (1999). Increased mortality, hypoactivity, and hypoalgesia in cannabinoid CB1 receptor knockout mice. Proc. Natl. Acad. Sci. USA.

[14] Buckley N.E., McCoy K.L., Mezey E., Bonner T., Zimmer A., Felder C.C., Glass M. (2000). Immunomodulation by cannabinoids is absent in mice deficient for the cannabinoid CB(2) receptor. Eur. J. Pharmacol..

[15] Ravinet Trillou C., Delgorge C., Menet C., Arnone M., Soubrié P. (2004). CB1 cannabinoid receptor knockout in mice leads to leanness, resistance to diet-induced obesity and enhanced leptin sensitivity. Int. J. Obes. Relat. Metab. Disord..

[16] Osei-Hyiaman D., Liu J., Zhou L., Godlewski G., Harvey-White J., Jeong W.-I., Bátkai S., Marsicano G., Lutz B., Buettner C. (2008). Hepatic CB1 receptor is required for development of diet-induced steatosis, dyslipidemia, and insulin and leptin resistance in mice. J. Clin. Investig..

[17] Deveaux V., Cadoudal T., Ichigotani Y., Teixeira-Clerc F., Louvet A., Manin S., Nhieu J.T.-V., Belot M.P., Zimmer A., Even P. (2009). Cannabinoid CB2 receptor potentiates obesity-associated inflammation, insulin resistance and hepatic steatosis. PLoS ONE.

[18] Agudo J., Martin M., Roca C., Molas M., Bura A.S., Zimmer A., Bosch F., Maldonado R. (2010). Deficiency of CB2 cannabinoid receptor in mice improves insulin sensitivity but increases food intake and obesity with age. Diabetologia.

[19] Alshaarawy O., Kurjan E., Truong N., Olson L.K. (2019). Diet-induced obesity in cannabinoid-2 receptor knockout mice and cannabinoid receptor 1/2 double-knockout mice. Obesity.

[20] Ruiz de Azua I., Lutz B. (2019). Multiple endocannabinoid-mediated mechanisms in the regulation of energy homeostasis in brain and peripheral tissues. Cell. Mol. Life Sci..

[21] Shin H., Han J.H., Yoon J., Sim H.J., Park T.J., Yang S., Lee E.K., Kulkarni R.N., Egan J.M., Kim W. (2018). Blockade of cannabinoid 1 receptor improves glucose responsiveness in pancreatic beta cells. J. Cell. Mol. Med..

[22] Lenzen S. (2014). A fresh view of glycolysis and glucokinase regulation: History and current status. J. Biol. Chem..

[23] Thorens B. (2015). GLUT2, glucose sensing and glucose homeostasis. Diabetologia.

[24] Kallendrusch S., Hobusch C., Ehrlich A., Ziebell S., Ueda N., Geisslinger G., Koch M., Dehghani F. (2012). Site-specific and time-dependent activation of the endocannabinoid system after transection of long-range projections. PLoS ONE.

[25] Bazwinsky-Wutschke I., Bieseke L., Mühlbauer E., Peschke E. (2014). Influence of melatonin receptor signalling on parameters involved in blood glucose regulation. J. Pineal Res..

[26] Tharp W.G., Lee Y.-H., Maple R.L., Pratley R.E. (2008). The cannabinoid CB1 receptor is expressed in pancreatic delta-cells. Biochem. Biophys. Res. Commun..

[27] Juan-Picó P., Fuentes E., Bermúdez-Silva F.J., Javier Díaz-Molina F., Ripoll C., Rodríguez de Fonseca F., Nadal A. (2006). Cannabinoid receptors regulate Ca^2+^ signals and insulin secretion in pancreatic beta-cell. Cell Calcium.

[28] Bermudez-Silva F.J., Sanchez-Vera I., Suárez J., Serrano A., Fuentes E., Juan-Pico P., Nadal A., Rodríguez de Fonseca F. (2007). Role of cannabinoid CB2 receptors in glucose homeostasis in rats. Eur. J. Pharmacol..

[29] Nakata M., Yada T. (2008). Cannabinoids inhibit insulin secretion and cytosolic Ca^2+^ oscillation in islet beta-cells via CB1 receptors. Regul. Pept..

[30] Cota D., Marsicano G., Tschöp M., Grübler Y., Flachskamm C., Schubert M., Auer D., Yassouridis A., Thöne-Reineke C., Ortmann S. (2003). The endogenous cannabinoid system affects energy balance via central orexigenic drive and peripheral lipogenesis. J. Clin. Investig..

[31] Chambers A.P., Sharkey K.A., Koopmans H.S. (2004). Cannabinoid (CB)1 receptor antagonist, AM 251, causes a sustained reduction of daily food intake in the rat. Physiol. Behav..

[32] Hildebrandt A.L., Kelly-Sullivan D.M., Black S.C. (2003). Antiobesity effects of chronic cannabinoid CB1 receptor antagonist treatment in diet-induced obese mice. Eur. J. Pharmacol..

[33] Röder P.V., Wu B., Liu Y., Han W. (2016). Pancreatic regulation of glucose homeostasis. Exp. Mol. Med..

[34] Bazwinsky-Wutschke I., Zipprich A., Dehghani F. (2017). Daytime-dependent changes of cannabinoid receptor type 1 and type 2 expression in rat liver. Int. J. Mol. Sci..

[35] Flake N.M., Zweifel L.S. (2012). Behavioral effects of pulp exposure in mice lacking cannabinoid receptor 2. J. Endod..

[36] Fattore L., Fratta W. (2010). How important are sex differences in cannabinoid action?. Br. J. Pharmacol..

[37] Wagner E.J. (2016). Sex differences in cannabinoid-regulated biology: A focus on energy homeostasis. Front. Neuroendocrinol..

[38] Cooper Z.D., Craft R.M. (2018). Sex-dependent effects of cannabis and cannabinoids: A translational perspective. Neuropsychopharmacology.

[39] Verty A.N.A., Stefanidis A., McAinch A.J., Hryciw D.H., Oldfield B. (2015). Anti-obesity effect of the CB2 receptor agonist JWH-015 in diet-induced obese mice. PLoS ONE.

[40] Jörns A., Tiedge M., Ziv E., Shafrir E., Lenzen S. (2002). Gradual loss of pancreatic beta-cell insulin, glucokinase and GLUT2 glucose transporter immunoreactivities during the time course of nutritionally induced type-2 diabetes in Psammomys obesus (sand rat). Virchows Arch..

[41] Frese T., Bazwinsky I., Mühlbauer E., Peschke E. (2007). Circadian and age-dependent expression patterns of GLUT2 and glucokinase in the pancreatic beta-cell of diabetic and nondiabetic rats. Horm. Metab. Res..

[42] Hinden L., Udi S., Drori A., Gammal A., Nemirovski A., Hadar R., Baraghithy S., Permyakova A., Geron M., Cohen M. (2018). Modulation of renal GLUT2 by the cannabinoid-1 receptor: Implications for the treatment of diabetic nephropathy. J. Am. Soc. Nephrol..

[43] Pingitore A., Ruz-Maldonado I., Liu B., Huang G.C., Choudhary P., Persaud S.J. (2017). Dynamic profiling of insulin secretion and ATP generation in isolated human and mouse islets reveals differential glucose sensitivity. Cell. Physiol. Biochem..

[44] Hinden L., Tam J. (2019). Do endocannabinoids regulate glucose reabsorption in the kidney?. Nephron.

[45] Matschinsky F.M., Wilson D.F. (2019). The central role of glucokinase in glucose homeostasis: A perspective 50 years after demonstrating the presence of the enzyme in islets of Langerhans. Front. Physiol..

[46] Matschinsky F.M. (2002). Regulation of pancreatic beta-cell glucokinase: From basics to therapeutics. Diabetes.

[47] Leibiger B., Leibiger I.B., Moede T., Kemper S., Kulkarni R.N., Kahn C.R., de Vargas L.M., Berggren P.O. (2001). Selective insulin signaling through A and B insulin receptors regulates transcription of insulin and glucokinase genes in pancreatic beta cells. Mol. Cell.

[48] Li C., Bowe J.E., Huang G.C., Amiel S.A., Jones P.M., Persaud S.J. (2011). Cannabinoid receptor agonists and antagonists stimulate insulin secretion from isolated human islets of Langerhans. Diabetes Obes. Metab..

[49] Flores L.E., Alzugaray M.E., Cubilla M.A., Raschia M.A., Del Zotto H.H., Román C.L., Suburo A.M., Gagliardino J.J. (2013). Islet cannabinoid receptors: Cellular distribution and biological function. Pancreas.

[50] Livak K.J., Schmittgen T.D. (2001). Analysis of relative gene expression data using real-time quantitative PCR and the 2(-Delta Delta C(T)) Method. Methods.

