# Supplementary material for: Influence of Cannabinoid Receptor Deficiency on Parameters Involved in Blood Glucose Regulation in Mice
Source: Int J Mol Sci. 2020 Apr 30;21(9):3168. doi: 10.3390/ijms21093168 (PMC7246639; doi:10.3390/ijms21093168)
Supplement: Supplementary file 1 [file ijms-21-03168-s001.pdf]

Table 1. Primers and sequences.

Gene	Forward primer	Reverse primer	Size (bp)	GenBank ID
mCB_1 (Cnr1)	ACAGGGCAGTACCCCTTCTT	AGCCCCTGGTGGTATTCTCT	175	NM_007726.3
mCB_2 (Cnr2)	TCATTGCCATCCTCTTTTCC	GAACCAGCATATGAGCAGCA	188	NM_009924.4
β-Actin (Actb)	ACTCCTACGTGGGCGACGAGG	CAGGTCCAGACGCAGGATGGC	389	NM_007393.5
mIns1	AGCAAGCAGGTCATTGTTTC	GGCGGGACTTGGGTGTGTAG	189	NM_008386
mIns2	TCCGCTACAATCAAAAACCAT	GCTGGGTAGTGGTGGGTCTA	410	AK007612
mGcg	ACCTGGACTCCCGCCGTG	TGGTGCTCATCTCGTCAGAG	279	BC012975
mSst	ATGCTGTCCTGCCGTCTC	TTCTCTGTCTGGTTGGGCTC	194	NM_009215
mGlut1 (Slc2a1)	AAAGAAGAGGGTCGGCAGAT	ACAGCGACACCACAGTGAAG	244	NM_011400
mGlut2 (Slc2a2)	GCCTGTGTATGCAACCATTG	GAAGATGGCAGTCATGCTCA	205	NM_031197
mGck	GCTTCACCTTCTCCTTCCCT	CATTCACCATTGCCACCACA	174	NM_010292.5

Table 2. Primary and secondary antibodies for immunohistochemistry.

Primary antibodies	Host	Dilution	Secondary antibodies
Anti-Glut1 (SPM498, monoclonal) MA5-11315 [a]	Mouse	1:10	Goat anti-mouse-Cy3[g]
Anti-Glut2 (polyclonal) 07-1402-I [b]	Rabbit	1:200	Goat anti-rabbit-Cy3[g]
Anti-glucokinase (polyclonal) (in house) [c]	Rabbit	1:50	Goat anti-rabbit-Cy3[g]
Anti-CB_1 (polyclonal) GP-Af530 [d]	Guinea pig	1:10	Goat anti-guinea pig-Alexa Fluor 488[f]
Anti-glucagon (K79bB10, monoclonal) G-2654 [e]	Mouse	1:100	Goat anti-mouse-DTAF[g] or Goat anti-mouse-Alexa Fluor 568[f]
Anti-glucagon (polyclonal) STJ23760[f]	Rabbit	1:200	Goat anti-rabbit-Cy2[g]

[a,f]Thermo Fisher Scientific, Waltham, MA, USA; [b]Merck Millipore, Darmstadt, Germany; [c]kindly provided by Anne Jörns, Institute of Clinical Biochemistry, Hannover, Germany; [d]Frontier Institute Co.Ltd., Hokkaido, Japan; [e]Sigma-Aldrich, Taufkirchen, Germany; [f]St John`s Laboratory Ltd, London, UK; [g]Dianova GmbH, Hamburg, Germany
